# Care for the Caregiver: How Caregiver Mortality Affects Treatment Outcomes—An Observational Cohort Study

**DOI:** 10.1007/s10461-025-04760-5

**Published:** 2025-05-20

**Authors:** Josephine Keal, Nicola van Dongen, Thalia Ferreria, Gillian Sorour, Karl-Günter Technau

**Affiliations:** Empilweni Services and Research Unit, Department of Paediatrics and Child Health, School of Clinical Medicine, Faculty of Health Sciences, Rahima Moosa Mother and Child Hospital, University of the Witwatersrand, Johannesburg, South Africa

**Keywords:** HIV/AIDS, Caregiver, Mortality, Orphan

## Abstract

Despite improvements in HIV management, children and adolescents living with HIV remain vulnerable. Caregiver mortality in a large paediatric and adolescent HIV clinic in Johannesburg is described and the effect of the death of a caregiver on children and adolescents’ HIV treatment outcomes was investigated. We analysed retrospective longitudinal data and included children or adolescents attending the clinic between 01 January and 31 December 2021 divided into those with documented primary caregiver mortality and those without (ever documented). Viral load, treatment regimens, CD4, and anthropometry were analysed for 2021. Caregiver vital status was recorded in 1171 (93%) of the 1260 patients attending in 2021. In 115 children or adolescents (10%) we found a documented death of caregiver(s). Amongst 1120 mothers, 100 (9%) had died; of 460 fathers, 18 (4%) had died and one (1%) of 100 other caregivers had died. A large number (n = 54 [45%]) of the 119 deaths occurred between 2016 and 2021 and 66 (69%) after the child/adolescent’s enrolment in the clinic. In 2021, stunting and wasting were more common in the participants with caregiver death than those without (χ2 = 4.98, 6.64, p = 0.01 and 0.03 respectively). No significant difference was seen between the groups for viral load, treatment regimens and CD4 counts. Caregiver death was incompletely captured in the clinic database, suggesting that clinicians were unaware of the death of a caregiver. Children experiencing the death of a caregiver were more likely to be malnourished. We propose increasing attention on the wellbeing of caregivers in paediatric HIV services.

## Introduction

There have been significant advancements in paediatric and adolescent human immunodeficiency virus (HIV) care since the roll-out of antiretroviral therapy (ART), especially so in sub-Saharan Africa [1]. However, progress in paediatric HIV care continues to lag behind adults, with 77% of eligible adults receiving ART globally, compared with 57% of children in 2022 [2]. South Africa now has the largest ART programme in the world, providing treatment to roughly 5.7 million people [3]. Despite this success, according to The Joint United Nations Programme on HIV and Acquired Immunodeficiency Syndrome (*UNAIDS*) only 54% of children known to be living with HIV in South Africa were on ART in 2022 [4].

In 2022 HIV/AIDS related disease resulted in 13.9 million children being orphaned globally [5]. The after-effects of HIV-associated mortality in the 1990s and early 2000s are still being felt. In 2020 there were 2.9 million recorded orphans in total in South Africa. This is equivalent to approximately 14% of all children and adolescents living in South Africa [6]. An orphan is defined as a child or adolescent who has experienced the death of either their biological mother or father or both. Paternal orphans (biological father demised) make up 61% of orphans in South Africa in 2020 [6]. The total number of orphans increased by over a million between 2002 and 2009 in South Africa and was largely attributed to deaths related to the HIV/AIDS pandemic, after which the trend was reversed [6]. Improved access to antiretrovirals resulted in orphan numbers dropping to lower than 2002 levels by 2017 [6]. There has been a 70% decrease in the number of people dying of HIV each year in South Africa since 2010 but despite this improvement, it was estimated that there were 720 000 orphans (between the ages of 0–17 years) living in South Africa in 2022 due to one of their parents dying from AIDS. [6]

Adherence to prescribed treatment regimens is a necessity for effective long-term therapy on ART. Recent studies suggest that adherence levels need to range from 70 to 90% for treatment regimens to be effective [7]. Children and adolescents living with HIV have unique barriers to achieving optimal adherence as they are often placed on ART from a very young age, and face the prospect of being on treatment for life [8]. The majority (90%) of children and adolescents living with HIV reside in sub-Saharan Africa [1]. Therefore, in addition to the challenges faced with pill burden, tolerability and acceptance of a diagnosis of HIV infection, these children are faced with the myriad of daily struggles associated with living in a lower middle-income setting. A primary caregiver is defined by the South African Social Security Agency as the person responsible for a child’s daily needs and well-being [9]. Caregivers living with HIV battle with their own adherence, acceptance and social challenges and it is not uncommon for caregivers to die from HIV/AIDS-associated illness [10]. The death of a primary caregiver can equate to the loss of a provider for the child and as a result; food security, shelter and other basic needs may be compromised. The psychological trauma of the death of a primary caregiver also negatively impacts the overall well-being of the child and the child is more vulnerable to abuse and neglect [10].

In Sub-Saharan Africa, up to 50% of children orphaned from HIV/AIDS who are living with HIV are now adolescents [11]. These children and adolescents are often cared for by HIV uninfected extended family members. Data suggest that caregivers of orphaned children and adolescents may stigmatise or discriminate against these children due to their HIV status, and this may result in poorer clinical outcomes [11].

With the above in mind, this study aimed to evaluate and describe a paediatric population attending a specialised HIV clinic in Johannesburg focusing on the primary caregivers of these patients. Particularly, this study analysed how the death of a caregiver impacted treatment outcomes such as viral load suppression rates, treatment regimens, CD4 counts and anthropological measurements to assess the health and well-being of the children and adolescents included.

## Methods

We conducted a retrospective analysis of longitudinal data collected during routine clinical visits at the Empilweni Services and Research Unit (ESRU), a paediatric and adolescent HIV clinic based at Rahima Moosa Mother and Child Hospital (RMMCH) in Johannesburg, South Africa.

The ESRU clinic, located within RMMCH- an urban academic hospital specialising in obstetrics, gynaecology, paediatric, and neonatal services- is a large paediatric HIV clinic in South Africa. Since it’s inception in 1998 there have been over 5,000 patients managed in total and 1,400 children are in active care. The clinic provides comprehensive care for neonates, children, and adolescents living with HIV, as well as HIV-exposed but uninfected neonates. On average, each patient visits the clinic four times per year. The clinical management includes initiation of first-line antiretroviral therapy (ART) for treatment-naïve patients, regular follow-up for patients established on ART, and management of patients with suspected or confirmed ART drug resistance, including regimen adjustments and adherence support. At clinic entry, demographic information is recorded for both the patient and their primary caregiver, including caregiver relationship to the child (e.g., biological parent, extended family member, institutional caregiver), caregiver age, national identity or passport number (if available), HIV status, ART regimen, and details of other children in their care. Caregiver and escort (if the child is accompanied by someone other than a primary caregiver) details are updated at each visit.

During follow-up visits, patients are seen by doctors and counsellors, and blood tests are performed by phlebotomy staff. Viral load (VL) monitoring follows national guidelines: at three months post-ART initiation, six months later if suppressed (VL < 50 copies/mL), and annually thereafter if suppression is maintained [12]. Mental health screening is conducted at every routine visit, with intensified screening for adolescents [13]. Until a recent hospital policy change, caregivers of children receiving treatment at the clinic were unable to receive their own ART treatment at RMMCH; making a family-centered approach to care very difficult.

This study focused on all children and adolescents under 20 years of age who attended the clinic between January 1, 2021, and December 31, 2021, regardless of their original enrolment date. The cut-off age of 20 years was chosen to include all adolescents receiving care at the clinic. Patients may have been enrolled prior to and including 2021. Data were collected on this cohort from the clinic’s inception in 1998 to the end of 2021. Data were extracted from the clinical visit database hosted on the University of the Witwatersrand REDCAP platform [14]. There was no sampling for this study, i.e. all available data of children and adolescents attending were included to ensure that a complete picture of data availability and outcomes was achieved.

Data were routinely collected from patient hospital records and entered the database after each clinic visit. Extracted variables included caregiver characteristics such as demographics (age, relationship to child), vital status at the child’s clinic entry and during follow-up, and caregiver mortality (with documentation source: hospital record or national death registry). Child and adolescent characteristics included age at clinic entry, mode of entry (via vertical transmission prevention [VTP] program or in-/outpatient services), age in 2021, VL suppression status (categorized as < 50, 50- < 1000, or ≥ 1000 RNA copies/mL), ART regimen type (Integrase Strand Transfer Inhibitors [INSTI], Protease Inhibitors [PI], or Non-Nucleoside Reverse Transcriptase Inhibitors [NNRTI]-based), WHO clinical stage at entry, nadir CD4 count, anthropometric measurements (weight-for-age, height-for-age, and weight-for-height Z-scores for children < 5 years; BMI Z-scores for those > 5 years), ART initiation date, and clinic visit frequency in 2021.

Caregiver mortality analysis included deaths categorized by caregiver type (mother, father, or other primary caregiver) and classified by documentation method. The timing of deaths was analyzed in relation to the child’s clinic entry, caregiver and child’s age at the time of death, and time-period (pre-2005, 2006–2010, 2011–2015, 2016–2021). Data were stratified into groups based on caregiver mortality status to assess differences in clinic entry and 2021 outcomes.

Continuous variables were summarised using medians and interquartile ranges (IQR). Categorical variables were presented as frequencies and percentages and compared using Chi-square or Fisher’s exact tests, depending on sample size. The Student’s t-test was used for normally distributed continuous variables, while the Wilcoxon test was applied to non-normally distributed variables.

Ethical clearance was obtained from the University of the Witwatersrand Human Research Ethics Committee (M220516). The cohort has been prospectively captured under the study protocol since 2007: *Cohort Study of HIV-infected and exposed Children receiving care at Rahima Moosa Mother and Child Hospital supported by the Empilweni Services and Research Unit and participation in the International epidemiological Databases to Evaluate AIDS (IeDEA) Collaboration* (M220559). Ethical approval includes annual data matching to the national death registry via the Medical Research Council, a trusted third party authorised to handle registry data for research purposes.

All data were stored in a password-protected, de-identified spreadsheet accessible only to the researcher and supervisor. Since 2007, all patients and caregivers entering the clinic have been invited to provide data-sharing consent.

## Results

**Fig. 1:**
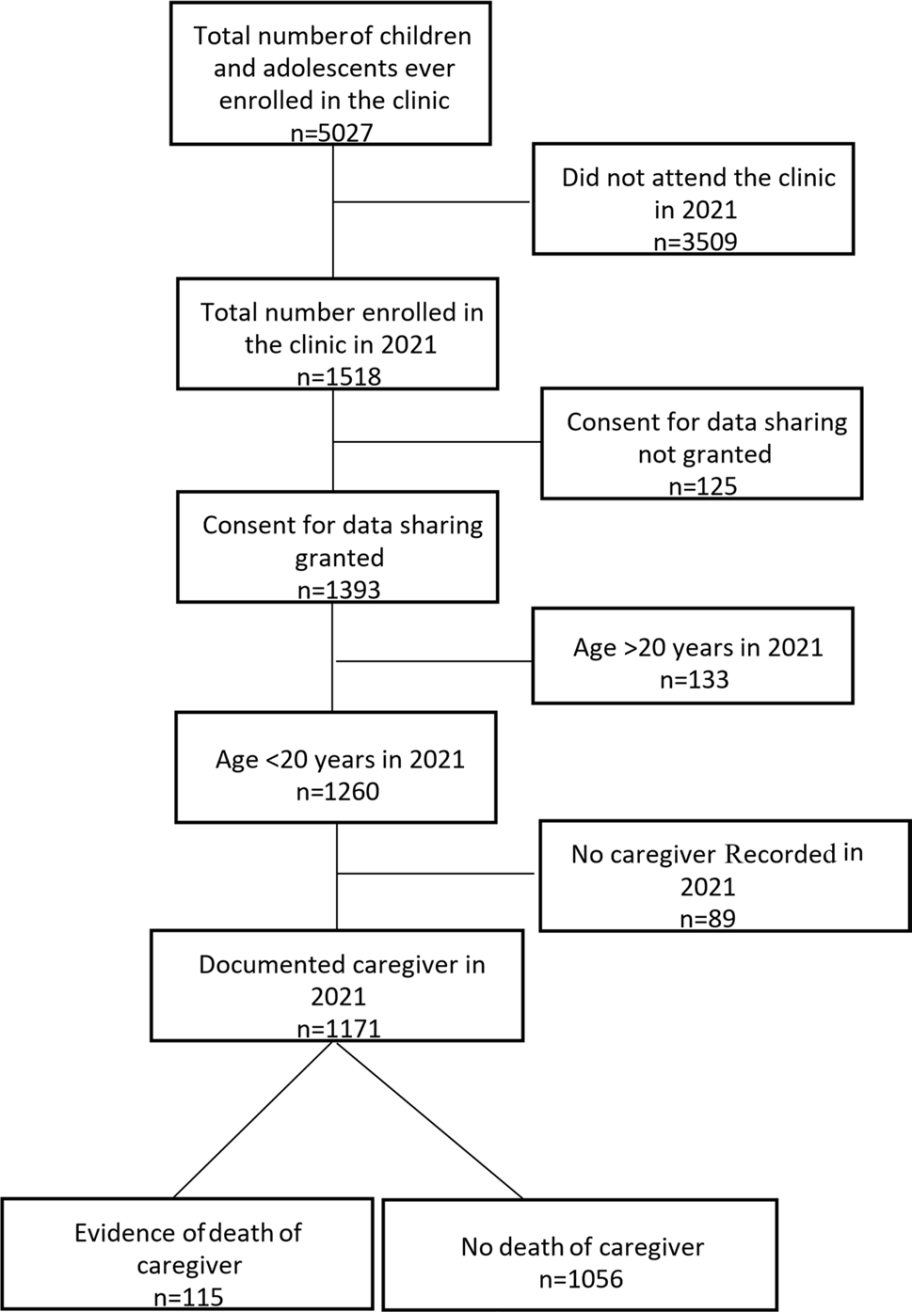
A flow diagram showing the process of screening potential participants attending a specialised paediatric and adolescent HIV clinic including the vital status of their caregiver in 2021

A total of 1518 children and adolescents attended the clinic during the study period. For 89 (6%) patients, there was no record of a caregiver in the clinic data system. After exclusions (Fig. 1) 1171 patients were included in the study. Caregiver status at entry to the clinic included 1120 (96%) mothers, 460 (39%) fathers and 100 (7%) other caregivers including 28 grandmothers, 48 other family members (aunts, uncles and siblings), 11 adoptive or foster parents and 13 other caregivers. In those cases where a biological mother was the documented primary caregiver, only 37% had a biological father who was an additional or secondary caregiver.

There were 115 (10%) children or adolescents with a documented death of caregiver(s) (Fig. 1). Amongst 1120 mothers, 100 (9%) had died; of 460 fathers, 18 (4%) had died and one (1%) of 100 other caregivers had died. There were four children or adolescents who experienced the death of two caregivers. In three cases both parents had died, and in one case the mother and another caregiver (other than the father) had died.

Out of the 1120 mothers, 1037 had a documented date of birth and their age in 2021 could be calculated. The median age of caregivers who were mothers attending the clinic in 2021 was 39.6 years (IQR: 34–44; Table 1). For 66 of the 100 mothers who died, both the date of birth and death were known and the median age at death was 35.2 years (30–40). Of the 460 fathers, 314 (68%) had a documented date of birth and their age in 2021 was 44.3 years (3849). Ten of the 18 fathers who had died had both date of birth and date of death and were 43.2 years (33–52) at the time of death.

**Table 1:** Ages of caregiver and children and relation to clinic entry in cases with caregiver death

	All	Mother	Father	Other
Total	119	100	18	1
Median age of child (years) at time of death (IQR)	6.9 (3.5–11.7) N = 95	6.1 (3.1–10.3) N = 81	10.6 (5.113.5) N = 13	12.7 N = 1
Median age of caregiver (years) at time of death (IQR)	35.3 (30.241.7) N = 77	35.2 (29.939.2) N = 66	43.2 (32.752.1) N = 10	65.4 N = 1
Death in relation to entry into clinic N (%)				
After entry	66 (69)	57 (70)	8 (62)	1 (100%)
Same year as entry	10 (11)	8 (10)	2 (15)	0
Before entry	19 (20)	16 (20)	3 (23)	0
Unknown	24 (20)	19 (20)	5 (28)	0
Year of death N (%)				
2005 and earlier	2 (2)	2 (2)	0	0
2006–2010	13 (11)	13 (13)	0	0
2011–2015	23 (19)	19 (19)	4 (22)	0
2016–2021	54 (45)	44 (44)	9 (50)	1 (100)
Unknown	27 (23)	22 (22)	5 (28)	0

*IQR* Inter-quartile range

For 78 of the 100 mothers who died the year of death was known and 44 (56%) of these deaths occurred between 2016 and 2021. Similarly, out of 13 of the 18 fathers with known year of death, 9 (69%) died between 2016 and 2021. Of 44 mothers who died between the years 2016–2021, 35 (80%) died while their children were enrolled at the clinic. Similarly, 7 (78%) of the 9 fathers who died between 2016 and 2021 had a child enrolled at the clinic. Overall, there were significantly more deaths (43/54 [80%]) that occurred while the patient was enrolled from 2016 to 2021 compared to before 2016 where 20/38 (53%) died after the child had already been enrolled (χ2 = 7.56, p = 0.023).

The clinic database as well as the national death registry were cross-referenced to attain the total number of deaths of caregivers per study participants. The clinic data recorded 87 deaths in caregivers who were mothers. When looking at the National Death Registry (NDR) a total of 40 (40%) maternal deaths were captured. Twenty-seven (27%) of these were captured in both databases, 13 (13%) were only captured on the NDR. Of the 18 deaths of fathers captured, 14 (78%) were captured in the clinic database. Five (28%) were captured in the national death registry and only one (6%) was captured in both databases. One death was recorded in a primary caregiver other than a mother or father, and it was noted in both databases.

Most of deaths occurred between 2016 and 2021, with 20 (20%) occurring in 2019.

There is a steady increase in the age of the child when the caregiver died from 2006 to 2021. The mean age of the child at the time of the death of the caregiver in 2005 was 2.0 years (1.5-2.0), versus 2016–2021 where it was 9.2 years (5–13; p < 0.0001 Wilcoxon test).

Table 2 shows the comparison between children and adolescents attending clinic in 2021 who had (n = 115) and had not experienced the death of a caregiver (n = 1056). The table addresses both characteristics at entry into HIV care as well as characteristics in 2021.

**Table 2:** Characteristics of patients attending the clinic in 2021

Characteristics	Total	Caregiver mortality	No caregiver mortality	Statistic and value	P value
Total	1171	115 (10)	1056 (90)		
Demographics and characteristics at entry to HIV services					
Female sex	579 (49)	54 (47)	525 (49)	χ^2^ = 0.44	0.80
Entry into clinic					
VTP	133 (11)	4 (3)	129 (12)	χ^2^ = 11.46	0.0095
Inpatient	492 (42)	49 (43)	443 (42)		
Outpatient	526 (45)	62 (54)	464 (44)		
Not known	20 (2)	0 (0)	19 (2)		
Median age in years at first visit (IQR)	2.3 (0.6–5.5)	3.0 (1.0–6.4)	2.1 (0.5–5.2)	t = − 2.26	0.026
Median weight for age zScore^§^ (IQR)	− 1.6 (− 2.6–− 0.6)	− 1.8 (− 2.8–− 0.7)	− 1.5 (− 2.6–− 0.6)	t = 1.12	0.26
Median height for age zScore^§^ (IQR)	− 1.8 (− 2.8–− 0.7)	− 2.0 (− 3.0–− 0.9)	− 1.7 (− 2.8–− 0.7)	t = 1.65	0.10
Median body mass index for age z-Score^§^ (IQR)	− 0.6 (− 1.6–0.4)	− 0.8 (− 1.6–0.25)	− 0.6 (− 1.6–0.3)	t = 1.10	0.27
Median nadir CD4 count (IQR)¶	500(302–722)	502 (345–719)	512 (310–731)	t = 0.37	0.71
Median nadir CD4% (IQR)	26(15–35)	27 (16.4–34.0)	27 (15.4–35.0)	t = 0.12	0.90
WHO stage not documented	443(55)	45(52)	398(56)	χ^2^ = 10.92	0.09
WHO stage 1	130 (16)	11(13)	119(17)		
WHO stage 2	44 (5)	1(1)	43(6)		
WHO stage 3	133 (17)	19(22)	114(16)		
WHO stage 4	51 (6)	10(12)	41(6)		
Ever had tuberculosis	398 (34)	50(43)	348(33)	χ^2^ = 5.00	0.08
Antiretroviral treatment					
On ART at first visit (%)	330 (29)	33 (31)	297 (29)	χ^2^ = 0.17	0.68
Median age (years) at ART start (IQR)	1.6 (0.4–4.3)	2.1 (0.6–6.3)	1.4 (0.4–4.0)	t = − 2.44	0.016
Characteristics in 2021					
Median age in years in 2021 (IQR)	13.9 (9.6–16.6)	14.5 (10.6-–16.9)	13.5 (9.0–16.2)	t = − 2.49	0.013
Age group					
0- < 5 years	125 (11)	4 (3)	121 (11)	χ^2^ = 8.42	0.038
5- < 10 years	200 (17)	20 (10)	180 (17)		
10- < 15 years	410 (35)	39 (34)	371 (35)		
15- < 20 years	436 (37)	52 (45)	384 (36)		
Viral load (copies/ml) in 2021					
0- < 50	882 (75)	91 (79)	791 (75)	χ^2^ = 0.98	0.61
50–1000	150 (13)	14(12)	136(13)		
> 1000	86 (8)	10(9)	121(12)		
Regimen in 2021					
INSTI-based	913 (83)	96(83)	817(78)	χ^2^ = 4.68	0.19
NNRTI-based	16(1.2)	3(3)	13(1)		
Other/unknown	23(2)	2(2)	21(2)		
PI-Based	216(18)	14(12)	202(19)		
Anthropometric measures in 2021					
Weight for age z-Score < -2	51(18)	6 (27)	45 (17)	χ^2^ = 1.52	0.21
Height for age z-Score < -2	212 (22)	31(31)	181(21)	χ^2^ = 4.98	0.03
Body mass index for age z-Score < -2	79 (8)	15 (15)	64 (7)	χ^2^ = 6.64	0.01

*IQR* Inter-quartile range, *WHO* World Health Organisation, *χ*^*2*^ Chi-square statistic, *t* Student’s t-test statistic

All data reflected as n (%) unless otherwise indicated. § Z-Scores calculated according to the WHO 2007 standard calculated through the SAS. ¶CD4 count measured in µl/ml

At entry into the clinic, the most notable differences are that children and adolescents who had experienced death of a caregiver were slightly older (3.0 years vs. 2.1 years; t = − 2.26; p = 0.026). A smaller proportion of children and adolescents who experienced caregiver mortality (3% vs 12%) entered the clinic via Vertical Transmission Prevention (VTP) services (which implies testing during routine immunisation or birth visits as opposed to sick child visits).

The cohort who had experienced the death of a caregiver(s) were not significantly more underweight for age in 2021 than the cohort who had not experienced the death of a caregiver(s). Stunting was significantly higher in the cohort who had a caregiver death (χ2 = 4.98; p = 0.03) and a higher rate of wasting was seen in the participants who had a caregiver die than those who had not (χ2 = 6.64; p = 0.01).

There was no statistically significant difference between the two groups for rates of viral load suppression (power 69%), nadir CD4 count, ever having had TB and ART regimen in 2021.

## Discussion

Our study showed that nearly 10% of the clinic population in 2021 had experienced the death of a primary caregiver, and most deaths occurred while the child was in care at the clinic. Children, and to some degree adolescents, are dependent on the commitment and reliability of their caregiver to ensure adherence to their ART [15]. With the improvements in ART, HIV has now changed into a chronic condition. Chronic medical conditions have unique challenges for the caregivers of children living with them [16]. In a Ugandan cross-sectional from 2021 where caregiver burden for a child with HIV was described as the emotional, physical, financial, and social toll of caring for a child with a chronic illness, a high care burden was common [10]. The recommendation was to implement social and spiritual support systems that would help to support caregivers with the unique struggles experienced while caring for orphaned children living with HIV [10].

The health of the parent(s) or caregivers is integral to the health of the child. We noted that the caregiver death was incompletely captured and documented in the clinic database as 13% of the maternal deaths and 5% of the paternal deaths were only noted in the National Death Registry and not in the clinical notes or database; suggesting that the clinicians were unaware of the death. The year with the highest number of deaths was 2019 with 20% occurring in this year. The reason for this high number is unclear but could be related to increased awareness due to a mental health screening process being added to the clinic at this time. During the time of death of a caregiver, there is an element of social disruption as a newly allocated caregiver would have to take over these responsibilities. What is often overlooked is the bereavement process that the child needs to go through with the death of a primary caregiver, and how that may influence their adherence to ART. The death of a caregiver should prompt interventions to protect the child/adolescent from other adverse outcomes that may be secondary to the death of a caregiver.

In research done on the outcomes of children and adolescents orphaned by HIV, it has been shown that they have increased mortality and morbidity, regardless of their own HIV status [15]. In our study we showed that the cohort who had a primary caregiver death had significantly higher rates of wasting and stunting, suggesting chronic illness and /or malnutrition. Possible reasons for this could be worsening food insecurity (resulting in malnutrition) or poor adherence to ART (resulting in chronic illness), both of which could be due to the death of a primary caregiver and breadwinner. A cross-sectional study done in 2020 showed a direct correlation between improved quality of life and being a part of a nuclear family (family of two parents and their children) [16]. The death of one or both parents is associated with an increased risk of short and long-term mental health problems [11].

There was no difference in virological outcomes between the two cohorts, implying that the observed increase in wasting and stunting rates was likely due to socioeconomic issues rather than adherence issues. Addressing quality of life and mental health in relation to the death of a caregiver needs to be explored in future research.

Most of both maternal (67%) and paternal (80%) deaths occurred in the last five years of the observation period. This is counterintuitive and in stark contrast to the decline in orphanhood observed in society [6]. This raises concern for treatment fatigue and missed opportunities in addressing the health issues of the parents as the majority of the caregivers were attending the clinic with their child(ren) at the time of their demise, supporting the need for a family-centred approach to HIV care [17]. Preventing deaths amongst caregivers should be prioritised. Screening for treatment interruption, mental health issues as well as other noncommunicable diseases (e.g. PAP smears, screening for diabetes and high blood pressure) could be easily offered and have been started at our clinic. It will be important to monitor the trend in the future to assess whether these interventions have helped.

Within the cohort who had a primary caregiver death, children were slightly older at entry into the clinic. They were therefore less likely to be diagnosed through routine HIV testing offered during VTP services. This suggests that children may not have had the same access to routine early infant HIV diagnosis. The median age at starting ART was nine months later in the group of children who had a caregiver’s death. This delay is significant for children where the risk of mortality in the first years of life is around 50% in the absence of ART [18]. There may be different reasons for this later ART start. Mothers who struggled with acceptance of their own diagnosis or treatment may have been at risk of not accessing VTP services, as well as being at risk of poorer outcomes for themselves. Alternatively, the maternal HIV results may have been kept secret and may not have been known until the mother died and the child came into the care of a different caregiver. Our data did not allow us to understand this in greater detail and more in-depth analyses of the journeys of children whose parents, particularly whose mothers, died may help to understand this better. In a study done in 2015, childhood mortality was shown to rise 7–11 months before the mother’s death [19]. This is likely due to the mother being ill and being unable to provide adequately for the child, however, once the mother or primary caregiver has passed away another caregiver steps in and fulfils that role.

Speaking about the death of biological parents and other caregivers comes with its challenges. Dealing with death and bereavement in children and adolescents is not easy, whether the death happened long ago or recently [20]. The death of a primary caregiver should be seen as a defining event in a child or adolescent’s life and should immediately trigger a higher level of awareness and caution about potential long-term clinical and psychosocial problems. As part of routine history-taking, the health of the primary caregiver and family should be enquired about and documented at each visit. The first step, however, is to quantify the problem and create more awareness.

The health of a child is dependent on the support and stability of a healthy caregiver. More stringent screening tools need to be put in place to intervene and help caregivers who are ill, and to create a stronger system of documenting deaths. In the event of a death, support networks and structures need to be put into place to help the children or adolescents grieve and cope with the loss. We see that the effects of a caregiver’s death are not short-lived but rather translate to chronic issues such as stunting and wasting. A small section in the Integrated Management of Childhood Illness (IMCI) booklet asks about maternal health when the child visits the clinic [21]. According to the WHO, IMCI is an integrated approach that focuses on the health and well-being of children, aiming to reduce preventable mortality, decrease illness and disability, and promote healthy growth and development of children under five years of age.

We argue that policy should move toward treating the primary caregiver and the child as a unit, screening the dyad at every routine clinic visit as well as during any presentation for medical care. In addition, parents and children should ideally be able to access their HIV care and other primary health care needs in the same clinic on the same day.

Strengths of the study included a large sample size as well as a long study period. The study also pooled data from multiple sources to get the most accurate representation of caregiver mortality. The weaknesses are that it was a single-centre study and only looked at those children and adolescents attending the clinic in 2021.There was a significant amount of missing data, along with several confounders that could have contributed to the child’s overall well-being. As this was a retrospective study it was challenging to identify a specific outcome related to caregiver mortality in a cohort of children and adolescents who had been in care for various durations. This made it difficult to do a multivariate analysis. The number of participants was not adequate to reach 80% power for describing a difference of > 10% viral suppression. The study also highlighted the incomplete documentation of caregiver health and mortality, and it is possible that deaths were missed. The National Death Registry (NDR) uses South African identity numbers, therefore the data gathered using the NDR will have significant omissions as caregivers without identify numbers or caregivers who are foreign nationals would not have been captured on this system. Caregivers are routinely reminded to update their documentation and there is annual review of the data to document and reduce gaps.

This study was unique in looking at the effects of the death of any primary caregiver, not just the mother. In South Africa this is particularly important as many children are raised by extended family members or other primary caregivers. The study also looked at chronic clinical outcomes such as stunting and wasting rather than mortality outcomes alone. Many children in South Africa come from backgrounds where their biological parents, for numerous reasons, are not their primary caregiver. The death of an extended family member or grandparent acting as a primary caregiver may have as profound an effect on a child’s health and wellbeing as the death of a biological parent. This may be easily overlooked if not enquired about in enough detail in our setting. There was only one documented death of a non-biological parent primary caregiver in our study. The documentation of these deaths may be a potential weakness in our study and something to enquire further about.

There is room for further investigation into how caregiver mortality affects other aspects of child and adolescent wellbeing including but not limited to mental health, developmental milestones and school or academic performance. There is a need for increased surveillance of mothers’ and caregivers’ health when children are admitted to hospital, to screen maternal and caregiver health, including noncommunicable diseases, and identify and address issues before discharging the child back into their care. This study hopes to highlight the importance of seeing the caregiver and child as a dyad rather than treating the child in isolation. Documentation of the death of a caregiver is equally important so that clinicians are sensitized to screen for potential issues that may arise in the ongoing care of the child.
